# Technological advances in cancer immunity: from immunogenomics to single-cell analysis and artificial intelligence

**DOI:** 10.1038/s41392-021-00729-7

**Published:** 2021-08-20

**Authors:** Ying Xu, Guan-Hua Su, Ding Ma, Yi Xiao, Zhi-Ming Shao, Yi-Zhou Jiang

**Affiliations:** 1 Key Laboratory of Breast Cancer in Shanghai, Department of Breast Surgery, Fudan University Shanghai Cancer Center, Shanghai, China; 2 Department of Oncology, Shanghai Medical College, Fudan University, Shanghai, China; 3 Institutes of Biomedical Sciences, Fudan University, Shanghai, China

**Keywords:** Tumour immunology, Tumour immunology

## Abstract

Immunotherapies play critical roles in cancer treatment. However, given that only a few patients respond to immune checkpoint blockades and other immunotherapeutic strategies, more novel technologies are needed to decipher the complicated interplay between tumor cells and the components of the tumor immune microenvironment (TIME). Tumor immunomics refers to the integrated study of the TIME using immunogenomics, immunoproteomics, immune-bioinformatics, and other multi-omics data reflecting the immune states of tumors, which has relied on the rapid development of next-generation sequencing. High-throughput genomic and transcriptomic data may be utilized for calculating the abundance of immune cells and predicting tumor antigens, referring to immunogenomics. However, as bulk sequencing represents the average characteristics of a heterogeneous cell population, it fails to distinguish distinct cell subtypes. Single-cell-based technologies enable better dissection of the TIME through precise immune cell subpopulation and spatial architecture investigations. In addition, radiomics and digital pathology-based deep learning models largely contribute to research on cancer immunity. These artificial intelligence technologies have performed well in predicting response to immunotherapy, with profound significance in cancer therapy. In this review, we briefly summarize conventional and state-of-the-art technologies in the field of immunogenomics, single-cell and artificial intelligence, and present prospects for future research.

## Introduction

Tumor cells exist with nearby cells in sophisticated community, which strongly affects how tumor cells grow, behave and communicate with other cells.^1,2^ Among these cells, immune cells are critical players, and many studies have proven that crosstalk between tumor cells and immune cells is bidirectional. Indeed, immune cells both promote and inhibit carcinogenesis, tumor progression, metastasis, and recurrence. Therefore, here we focus on the tumor immune microenvironment (TIME).^2,3^ And accordingly, promoting the transition from a pro-tumor to an anti-tumor effect to maximize the efficacy of anti-tumor immunity is a main goal of immunotherapy.^4,5^ Recent tumor immunotherapy strategies, such as immune checkpoint blockades (ICBs), cancer vaccines, and adoptive cell transfer (ACT) therapy, have shown unprecedented clinical efficacy.^6–12^ Nevertheless, in the face of therapeutic resistance and adverse effects, among others, their applications are hindered by the incomplete understanding of tumor immunity.

Despite achieving great advancements in exploring the mechanism of tumor-immune interplay, traditional techniques, such as western blotting (WB), coimmunoprecipitation (Co-IP), and real-time quantitative polymerase chain reaction (RT-qPCR), cannot provide a thorough landscape of the TIME. There is an urgent need for novel methods to characterize tumor immunological features in detail. Applying high-throughput technologies, such as genomics, transcriptomics, proteomics, epigenomics, cytomics, and informatics, to comprehensively understand tumor immunity has emerged as a brand-new discipline, i.e., tumor immunomics, providing novel insights for researchers.^13,14^ Next-generation sequencing (NGS) technologies greatly promote the development of immunogenomics, an important branch of immunomics. Furthermore, single-cell sequencing and artificial intelligence (AI) have ushered in a new epoch of tumor immunity in recent years. Due to the tremendous development of tumor immunology and bioinformatics, an increasing number of technologies and potential clinical implications are a matter of great concern.

In this review, we discuss the technological advances and clinical implications of immunomics in tumors to date, especially in the field of immunogenomics, single-cell, and AI.

## Brief introduction to the TIME

Over the past years, knowledge of tumors has undergone metamorphosis due to innumerous researchers’ efforts to achieve progress against tumors. The definition of tumors has also evolved from the mere aggregation of tumor cells to a complex organ-like structure composed of tumor cells, immune cells, fibroblasts, vascular endothelial cells, and other stromal cells in communities.^15–17^ Encompassing all structures in the organ, such as immune infiltration, vascular vessels, the extracellular matrix, etc. the tumor surrounding, which is also called the tumor microenvironment (TME), has been one of the hottest research topics in oncology.^18,19^ With the development of tumor immunity, the immune context of the TME, i.e., TIME, has been proven to play a decisive role in carcinogenesis, tumor progression, metastasis, recurrence, and potential therapeutic targets making it being the focus of our review.^20,21^

There are two main categories for the compositions of the TIME, i.e., immune cells and secreted factors, such as cytokines, chemokines, and growth factors. Regarding the former, the TIME contains extremely diverse subsets of immune cells, including T lymphocytes, B lymphocytes, natural killer (NK) cells, macrophages, dendritic cells (DCs), granulocytes, and myeloid-derived suppressor cells (MDSCs), among others.^22,23^ Normally, T cells, B cells, NK cells, and macrophages help inhibit tumor growth, while MDSCs and regulatory T cells (Tregs) tend to suppress anti-tumor immunity.^23,24^ However, available studies have confirmed that given the complex interactions with tumor cells, the specific role of immune cells could dynamically change and even become the exact opposite. For example, the anti-tumor function of CD8^ +^ T cells may be inhibited via the exhaustion of T cells, and after CTLA-4 blockade in glycolysis-low tumors, the functional destabilization of Treg cells towards interferon-γ-producing cells may promote anti-tumor immunity.^25^

**Fig. 1:**
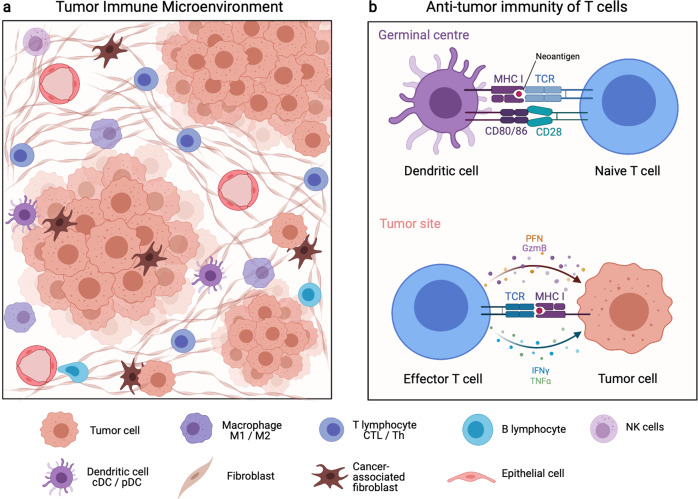
Components and interactions of the tumor immune microenvironment. **a** Cellular compositions of the tumor immune microenvironment. **b** Brief illustration of cell–cell interaction in anti-tumor immunity. cDC conventional dendritic cells, CTL cytotoxic T lymphocyte, Gzm granzyme, IFN interferon, MHC major histocompatibility complex, NK cells natural killer cells, pDC plasmacytoid dendritic cells, PFN perforin, TCR T cell receptor, Th T helper cell, TNF tumor necrosis factor

In summary, innumerable immune cell types and even different functional states of specific immune cell types may produce the opposite effect on anti-tumor immunity (Fig. 1). Thus, it is not wise to explore tumor immunity in a reductionistic way. With the aid of state-of-art bioinformatics technologies, to a great extent, researchers could characterize tumor immunological features systematically and provide more information to enhance our understanding of tumor immunity.

## Immunomics technologies in the NGS era—immunogenomics

Over the last two decades, NGS, including whole-genome sequencing (WGS), whole-exome sequencing (WES), and RNA sequencing (RNA-seq), has been successfully developed and applied to obtain whole-genome information in humans. Compared to Sanger sequencing, NGS generates high-throughput genomic and transcriptomic data, laying a foundation for research investigating the multi-step immune response. Studies utilizing immunogenomics in the NGS era not only provide a global view of the immune cell compositions of the TIME through bioinformatic algorithms but also identify immunogenic proteins by abnormal peptide prediction, human leukocyte antigen (HLA) typing, and major histocompatibility complex (MHC)-peptide binding affinity prediction.

### Quantification of immune cells in the TIME

The TIME comprises various immunocytes. For the quantification of tumor immune cell components in the TIME, conventional methods, such as flow cytometry and immunohistochemistry (IHC), are impractical for massive profiling because of their high cost and low tissue availability. With the rapid development of NGS, in silico analysis has become an alternative approach to address this issue. Considering the high cellular heterogeneity, gene expression profiles are very different among the different immune cell types and could represent immune cell types to a certain extent. Thus, we are able to estimate the abundance of dozens of immune cell types through NGS data, which have also been validated as reliable. The sources of these analyses are mainly DNA and RNA sequencing, especially the latter. Regarding RNA-seq data, which we mainly discuss, the rationales of the computational methods are mainly classified into gene set enrichment analysis (GSEA) and deconvolution^26^ (Tables 1–2).

**Table 1:** Computational tools used in tumor immunogenomics with high-throughput next-generation sequencing data

Tool	Characteristics	URL	Year	Ref.
*Quantification of immune cells in TIME*				
CIBERSORT	Based on linear support vector regression, deconvolution from microarray data, and known gene expression profiling set.	https://cibersort.stanford.edu/	2015	^30^
CIBERSORTx	Expanding data source to single-cell RNA-seq.	https://cibersortx.stanford.edu/	2019	^34^
DeconRNASeq	Constrained least square regression model validated with RNA-seq data from five human tissues	http://bioconductor.org/packages	2013	^273^
EPIC	Based on constrained least square, incorporates a non-negative condition into deconvolution.	https://gfellerlab.shinyapps.io/EPIC_1-1/	2017	^274^
ESTIMATE	Generating a stromal score and immune score to reflect the tumor purity based on ssGSEA	https://sourceforge.net/projects/estimateproject/	2013	^27^
FARDEEP	Based on adaptive least trimmed square, FARDEEP removes outliers and outputs the absolute quantification of cell types	https://github.com/YuningHao/FARDEEP.git	2019	^32^
MCP-counter	The score is the geometric mean of the expression level of cell-specific genes, implying the absolute abundance of immune cell types among samples	http://github.com/ebecht/MCPcounter	2016	^29^
MuSiC	Deconvolution of bulk sequencing data based on cell type-specific gene expression reference from single-cell RNA-seq	https://github.com/xuranw/ MuSiC	2019	^33^
NITUMD	A semi-supervised nonnegative matrix factorization framework with a trichotomous signature matrix	https://github.com/tdw1221/NITUMID	2020	^275^
PERT	A non-negative maximum likelihood-based method applied to fresh human umbilical cord blood samples	–	2012	^276^
quanTIseq	Quantification of ten different immune cell types and other uncharacterized cells based on RNA-seq data	http://icbi.at/quantiseq	2019	^277^
TIMER	Immune cells are estimated via transcriptomic data and the correlations among the immunological, genomic, and clinical features were established.	https://cistrome.shinyapps.io/timer/	2016	^278^
xCell	Spillover compensation is used to separate cell types with high correlation	http://xcell.ucsf.edu/	2017	^28^
*Prediction of mutated proteins*				
CN-Learn	Machine-learning framework integrating calls from multiple CNV detection algorithms and learning to accurately identify true CNVs	https://github.com/girirajanlab/CN_Learn	2019	^180^
deepSNV	Detecting and quantifying sub-clonal SNVs in mixed populations even for low-frequency variants	http://www.bioconductor.org	2012	^181^
DeepVariant	SNP and small-indel variant caller using deep neural networks in aligned NGS read data	https://github.com/google/deepvariant/	2018	^182^
EBCall	Discriminating somatic mutations from sequencing errors with both moderate and low allele frequencies	https://github.com/friend1ws/EBCall	2013	^61^
GATK	Industry standard for identifying SNPs and indels via analyzing WES, WGS, and RNA-seq data	https://gatk.broadinstitute.org/hc/en-us	2010	^51^
LoFreq	Modeling sequencing run-specific error rates to accurately call variants occurring in <0.05% of a population	http://sourceforge.net/projects/lofreq/	2012	^53^
MuTect2	Sensitive detection of somatic point mutations especially in low-allelic-fraction events	https://software.broadinstitute.org/cancer/cga/mutect	2013	^52^
Platypus	Using local de novo assembly to generate candidate variants, including SNPs, indels, and complex polymorphisms	http://www.well.ox.ac.uk/platypus	2014	^279^
PyroHMMsnp	Realigning read sequences around homopolymers and inferring the underlying genotype by using a Bayesian approach	https://github.com/homopolymer/PyroTools/	2013	^280^
SAMtools	Variant caller utilizing the post-processing alignments in the SAM/BAM format	http://samtools.sourceforge.net	2009	^62^
SCcaller	Firm foundation for standardized somatic-mutation analysis in single-cell genomics based on single-cell multiple displacement amplification (SCMDA)	https://github.com/biosinodx/SCcaller/	2017	^281^
SomaticSeq	Somatic mutation detection pipeline used to produce highly accurate somatic mutation calls for both SNVs and small INDELs	http://bioinform.github.io/somaticseq/	2015	^282^
SomaticSniper	Calling of somatic SNPs and indels from matched tumor–normal NGS data	http://gmt.genome.wustl.edu/packages/somatic-sniper/	2011	^56^
Strelka2	Fast and accurate caller of germline and somatic variants based on Strelka	https://github.com/Illumina/strelka	2018	^58^
VarDict	Variant caller of SNV, MNV, INDELs, and SVs, enabling ultra-deep sequencing	https://github.com/AstraZeneca-NGS/VarDict	2016	^60^
VarScan2	Detection of somatic mutations and CNVs in exome data from tumor–normal pairs	http://varscan.sourceforge.net	2012	^55^
*HLA typing*				
HISAT2	Graph-based genome alignment and genotyping, also applied for DNA fingerprinting	https://github.com/DaehwanKimLab/hisat2	2019	^84^
HLA-HD	Extraction of six-digit resolution HLA-I and HLA-II from NGS data	https://www.genome.med.kyoto-u.ac.jp/HLA-HD/	2017	^77^
HLA-miner	HLA-I and HLA-II typing directly from non-targeted RNA-seq, WGS and WES data	http://www.bcgsc.ca/platform/bioinfo/software/hlaminer	2012	^76^
HLAProfiler	K-mer profile-based method for HLA calling in RNA-seq data for both rare and common HLA alleles at two-field precision	https://github.com/ExpressionAnalysis/HLAProfiler	2017	^283^
HLAreporter	Extraction of HLA-I and HLA-II from NGS data at four-digit resolution	http://paed.hku.hk/genome/	2015	^81^
HLAscan	Determination of HLA type across the whole-genome, exome, and target sequences	http://www.genomekorea.com/display/tools/HLA_SCAN	2017	^284^
HLAssign	First highly automated open-source HLA-typing method for NGS data to three-field resolution	https://www.ikmb.uni-kiel.de/resources/download-tools/software/hlassign	2015	^285^
HLA-VBseq	Genotyping of HLA alleles at an 8-digit resolution from WGS data without the need of prior knowledge regarding the HLA loci	http://nagasakilab.csml.org/hla	2015	^79^
Kourami	Graph-guided assembly technique used to provide highly classical HLA typing	https://github.com/Kingsford-Group/kourami	2018	^85^
Optitype	Genotyping of major and minor HLA-I alleles from RNA-seq, WGS, and WES data not specifically enriched for the HLA cluster	http://github.com/FRED-2/OptiType	2014	^78^
PHLAT	High-accuracy genotyping of HLA-I and HLA-II alleles from RNA-seq, WGS, WES, and targeted sequencing at a four-digit resolution	https://sites.google.com/site/phlatfortype	2014	^83^
Polysolver	High-precision HLA typing of WES data even relatively low-coverage WES data, and subsequent mutation detection	http://www.broadinstitute.org/cancer/cga/polysolver	2015	^82^
seq2HLA	Using standard RNA-Seq reads as input to determine the HLA-I and HLA-II types and expression at a four-digit resolution	https://github.com/TRON-Bioinformatics/seq2HLA	2012	^70^
SNP2HLA	Imputing four-digit classical alleles and amino acid polymorphisms at class I and class II loci	http://faculty.washington.edu/browning/beagle/beagle.html	2013	^80^
*Prediction of antigen-MHC binding affinity*				
ACME	Pan-specific peptide–MHC class I binding prediction through attention-based deep neural networks	https://github.com/HYsxe/ACME	2019	^286^
MHCAttnNet	MHC-peptide binding prediction of MHC alleles classes I and II using an attention-based deep neural model	https://github.com/gopuvenkat/MHCAttnNet	2020	^287^
MHCflurry	Open-source class I MHC binding affinity prediction, using mass spectrometry datasets for model selection and showing competitive accuracy	https://github.com/openvax/mhcflurry	2018	^98^
MHCSeqNet	Open-source deep neural network model for universal MHC binding prediction, accepting peptides of any length	https://github.com/cmbcu/MHCSeqNet	2019	^288^
NetMHC	High accuracy prediction of pMHC binding affinity to human and non-human MHC-I molecules based on ANN and PSSMs	http://www.cbs.dtu.dk/ services/NetMHC	2008	^93^
NetMHCII	High accuracy prediction of pMHC binding affinity to human and non-human MHC-II molecules based on ANN and PSSMs	http://www.cbs.dtu.dk/services/NetMHCII	2018	^289^
NetMHCIIpan	Pan-specific version of netMHCII	http://www.cbs.dtu.dk/services/NetMHCIIpan	2020	^102^
NetMHCpan	Pan-specific version of netMHC	http://www.cbs.dtu.dk/services/NetMHCpan	2020	^102^
PSSMHCpan	PSSM based software for predicting class I peptide-HLA binding affinity	https://github.com/BGI2016/PSSMHCpan	2017	^94^
PUFFIN	Deep residual network-based computational approach that quantifies uncertainty in pMHC affinity prediction	http://github.com/gifford-lab/PUFFIN	2019	^290^

*ANN* artificial neural network, *CNVs* copy number variations, *HLA* human leukocyte antigen, *INDELs* insertions and deletions, *NGS* next-generation sequencing, *MNVs* multiple-nucleotide variants, *PSSM* position-specific scoring matrix, *RNA-seq* RNA sequencing, *SNP* single-nucleotide polymorphism, *SNVs* single nucleotide variations, *SVs* structural variants, *TIME* tumor immune microenvironment, *WES* whole-exome sequencing, *WGS* whole-genome sequencing

**Table 2:** Strengths and weaknesses of immune cells quantification algorithms

Algorithm	Category	Strengths	Weakness
ESTIMATE	G	Available for tumor purity and global immune status	Only a stromal score and an immune score are output. The information is limited^27^
xCell	G	Available for inference of 64 immune and stromal cell	The definitions of the cell subtypes are sometimes not clear Accuracy of prediction of some cell types is uncertain^26^
MCP-counter	G	Available for inference of fibroblasts and endothelial cells Available for an absolute quantification of specific cell population across samples Available for between-sample comparison	Relatively less cell types included in the inference (8 types)
CIBERSORT	D	Available for inference of 22 immune cell subtypes Available for between-cell-type comparison	Relative proportion of distinct cell types in a single sample Trained on microarray rather than RNA-seq data^30^
EPIC	D	Available for inference of fibroblasts, endothelial cells, and uncharacterized cells Enabling inference of tumor purity from uncharacterized cell proportion Available for both between-sample and between-cell-type comparison	Only 6 immune cell types available Not available for discrimination of cell types with transcriptional similarity
quanTIseq	D	Available for inference of 10 immune cell subtypes Available for both between-sample and between-cell-type comparison	Not available for quantification of stromal cells (e.g., cancer-associated fibroblasts)
TIMER	D	A user-friendly analytic web tool for cancer immunology research	Only 6 immune cell types and no stromal cells available Relative proportion of distinct cell types in a single sample
CIBERSORTx	D	Adopting a more convincing gene expression reference from single-cell sequencing	Suitability for some tumor types needs further validation
MuSiC	D	Adopting a more convincing gene expression reference from single-cell sequencing Available for tissues with intensively correlated cell types	Suitability for some tumor types needs further validation Not available for TPM data as input^33^
FARDEEP	D	A robust machine learning tool eliminating outliers in the dataset Suitable for deconvolution of noisy datasets	Different signature matrix should be adopted according to the type of gene expression data^32^

*G* GSEA-based method, *D* deconvolution method

In general, the representative GSEA-based algorithms include ESTIMATE, xCell, and MCP-counter. Based on the gene signature and single sample GSEA (ssGSEA), the ESTIMATE algorithm provides an immune score and a stromal score to represent the proportion and distribution of immune cells and stromal cells.^27^ The ESTIMATE score can differentiate the tumor and stomal components but cannot distinguish specific immune cell types. xCell is another ssGSEA-based method that obtains gene sets to characterize distinct cell types from multiple RNA-seq and microarray-based data sources, increasing the robustness to avoid noise disturbances. Compared with ESTIMATE, xCell uses a spillover compensation correction to better distinguish among cell types with close relationships and high similarity.^28^ MCP-counter generates an abundance score for each TIME cell population (including not only immune cells but also endothelial cells and fibroblasts) in every single sample based on the geometric mean of marker gene expression levels.^29^ For the sake of accuracy, a common characteristic of GSEA-based methods is the need for a specific gene set for each immunocyte subpopulation of interest.

The deconvolution of cell components is a reverse process of the convolution of cell subtypes in bulk tissues based on gene expression signatures. The deconvolution-based tools include DeconRNASeq, PERT, CIBERSORT, TIMER, EPIC, quanTIseq, and deconf.^26^ CIBERSORT, which is among the most popular algorithms based on deconvolution, utilizes linear support vector regression and a gene expression signature matrix to characterize immune infiltrating components.^30^ QuanTIseq is designed specifically for RNA-seq data, and the analysis pipeline comprises raw RNA-seq data preprocessing, gene expression quantification, and constrained least squares regression-based deconvolution. Remarkably, using this method, integrated image information from hematoxylin-eosin (H&E)-, IHC-, and immunofluorescence (IF)-stained slides is utilized to complement gene expression deconvolution, enabling immune profiling of the absolute cell fraction and unique immune cell densities.^31^ Recently, more novel deconvolution-based algorithms have been developed. For example, FARDEEP focuses on significant issue such that the deconvolution accuracy is influenced by outlier contamination of gene expression, which has not been addressed by previous algorithms.^32^ FARDEEP relies on the least trimmed square (LTS) to construct a robust model suitable for datasets with heavy-tailed noise. MuSiC considers cross-subject and cross-cell consistency and leverages cross-subject single-cell RNA sequencing (scRNA-seq) to generate cell type-specific gene sets for the deconvolution analysis of bulk RNA-seq data.^33^ However, the sensitivity and specificity of the newly developed algorithms mentioned above require more validation. Currently, ESTIMATE, CIBERSORT, and MCP-counter remain the most commonly used methods for determining immune components, and CIBERSORT has recently been updated to CIBERSORTx to fit single-cell sequencing data.^34^

These immunogenomics technologies are widely used to elaborate the global immune infiltration characteristics of specific cancer types. In recent multiomics studies, xCell was applied to portray the immune landscape of clear cell renal cell carcinoma, lung adenocarcinoma, and head and neck squamous cell carcinoma.^35–37^ Remarkably, an immune landscape was interpreted with 10000 tumors of 33 cancer types compiled in TCGA. Thorsson and colleagues divided the cancer-immune status into six distinct clusters, and CIBERSORT was used to dissect the composition of immune cells in each immune subtype.^38^ In addition, these algorithms were applied to compare the TIME composition of two or more groups of patients with distinct pathological features, therapeutic strategies, and treatment responses. Using CIBERSORT, Gil Del Alcazar et al.^39^ uncovered the difference in the infiltrating T cell subpopulation between breast ductal carcinoma in situ (DCIS) and breast invasive ductal carcinomas (IDCs). CD8^ +^ T cells were enriched in DCIS, whereas Tregs and CD4^ +^ T helper cells were more infiltrated in IDC. Wheeler et al. analyzed the components of the TME of hepatocellular carcinoma (HCC). These authors found that compared to normal adjacent tissue, HCC tissue was more likely to accommodate immunosuppressive cells. This research portrayed an immune evasion microenvironment and supported evidence suggesting that ICBs might be feasible in HCC patients with moderate to high levels of immune infiltration.^40^

Although RNA-seq data have been widely used as input resources for deconvolution, the instability of RNA molecules affects the accuracy of results obtained under chemical agent fixation. DNA molecules are more stable, and DNA methylation is highly cell-type specific, rendering DNA methylation a potential surrogate in TIME deconvolution. Cell composition dissection based on DNA methylation from blood samples has been reported, such as methylCIBERSORT and MethylResolver. The accuracy of methylCIBERSORT has been validated in immune infiltration analysis, and its clinical implications in both head and neck squamous cell carcinoma and pediatric central nervous system tumors have been presented.^41^ By adopting an LTS regression as described above using the FARDEEP algorithm, MethylResolver fabricates a methylation signature compendium of the leukocyte population and accomplishes relative quantification of immune infiltrates and evaluation of tumor purity.^42^

Notably, the combination of NGS data and bioinformatics algorithms could roughly differentiate immune cell types. Nevertheless, immune cells in the TIME comprise numerous subtypes with different properties and biological functions. Consequently, single-cell technologies should be developed to identify these cell subtypes at a higher resolution.

### Identification of tumor antigens

Genomic-level mutations, transcriptomic-level mutations, and proteomic-level alternations can cause the expression of abnormal proteins, i.e., tumor antigens, which can be recognized by immune cells and trigger the anti-tumor immune response.^43–45^ Among these antigens, viral antigens, cancer germline antigens, and neoantigens (tumor-specific antigens resulting from somatic DNA alterations) have relatively high tumoral specificity and, thus, have become the main tumor vaccine targets.^7,45–49^ Consequently, we mainly discuss the identification of these tumor-specific antigens, particularly neoantigens. According to the process of antigen recognition, immunogenomics technologies perform in silico analysis to predict abnormal peptides, perform HLA typing and predict MHC-peptide binding affinity, which is greatly helpful and necessary for the identification of tumor antigens (Table 1).

#### Prediction of abnormal peptides from WES, WGS, or RNA-seq data

Somatic DNA mutations, including single-nucleotide variants (SNVs) and small insertions and deletions (INDELs), account for the major sources of abnormal proteins.^45^ Given recent systematic reviews concerning variant detection tools, we provide a summary of several standard tools and briefly discuss future perspectives.^50^

Currently, Genome Analysis Toolkit (GATK) is the industry standard used to identify SNVs and INDELs by analyzing WES, WGS, and RNA-seq data. Its scope is also expanding to cover copy number variations (CNVs) and structural variations (SVs).^50,51^ In the case of variants with a low allele frequency (normally allelic fractions as low as 0.1 and below), LoFreq and MuTect have higher sensitivities with a similar specificity and may be a better choice; the latter, which applies a Bayesian classifier, has been used more widely.^52–54^ In contrast, VarScan2 and SomaticSniper require higher allele fractions to guarantee sufficiently high sensitivity.^55–57^ Regarding liquid tumor analysis, Strelka2 introduces a normal sample contamination model to improve the variant calling accuracy and functions fairly well in the computing cost.^58,59^ In addition, VarScan, FreeBayes, Samtools, Vardict, and EBCall are valuable for identifying tumor antigens.^55,60–62^

However, regarding false positivity or false negativity, none of these tools is satisfactory in all aspects. Therefore, the scientific community has not established gold standards for calling variants.^63,64^ How to optimize the present tools and design a versatile and efficient variant caller to better discriminate true variants from sequencing errors is worthy of further research.^65^ By integrating VarScan, GATK, Pindel, BreakDancer, Strelka, and Genome STRiP in a large web interface, the Genome Variant Investigation Platform (GenomeVIP) provides a new method and has been used in large data projects, such as TCGA PanCanAtlas, to provide high-confidence annotated somatic, germline, and de novo variants of potential biological significance.^66,67^ Furthermore, it is advisable to select more than two tools to predict abnormal proteins in practice.

#### HLA typing

Abnormal peptides need to bind HLA to assist recognition by the T cell receptor (TCR) to elicit an immune response. HLA genes are the most polymorphic genes in the human genome and comprise three major gene loci for class I (A, B, and C) and three for class II (DP, DQ, and DR).^68–71^ Different HLAs have distinct binding affinities to abnormal proteins. Thus, crucial for antigen recognition, predicting HLA typing is essential for the identification of tumor antigens.^70,72^

After a long development period, limited by their efficiency and reliability, serological and cellular typing methods have been gradually replaced by DNA typing methods.^73,74^ Although real-time polymerase chain reaction (PCR) and sequencing-based methods have been the standard HLA typing methods, their low throughput limits their wide application.^75^ In particular, the tools used for HLA typing in the era of NGS have dramatically changed the field. HLA-miner and Seq2HLA are two of the early tools used for HLA typing from NGS data, massively circumventing the time and cost at that time.^70,76^ Subsequently, great efforts have been achieved to improve HLA typing performance in terms of both accuracy and resolution. PHLAT, HLAreporter, SNP2HLA, HLA-HD, Optitype and HLA-VBSeq perform fairly well at a four-digit, six-digit, and eight-digit resolution in different cancers.^77–83^ Notably, among these tools, Polysolver enables high-precision HLA typing and is among the currently accepted standard tools using low-coverage WES data, particularly when applied to cancer-associated somatic mutations.^82^ Graph-guided genotyping tools used to perform highly classical HLA typing, such as Kourami and HISAT2, provide a new perspective to improve the efficacy of typing.^84,85^ However, considering the complexity of the HLA types, we still expect independent benchmarking studies and more tools to be presented.

#### Prediction of antigen-MHC binding affinity

In addition to identifying abnormal peptides and HLA typing, antigen-MHC binding affinity is the next focus of tumor antigen prediction.^86,87^ Human MHC molecules are divided into the following three subtypes: Class I, Class II, and Class III. Class I MHC molecules (MHC-Is) are expressed by all nucleated cells and present intracellular peptides, such as viral and tumor antigens, to CD8^ +^ T cells to elicit an immune response. In addition, expressed on professional antigen-processing cells (APCs), such as DCs, macrophages, and B cells, class II MHC molecules (MHC-II) present exogenous peptides to activate CD4^ +^ T cells.^88,89^ Despite substantial research on MHC-I and tumor immunotherapy, recent studies have shown that tumor-specific MHC-II molecules are also associated with favorable outcomes in patients with cancer.^90^ MHC IIIs are not markers on the cell surface and are not discussed here.

Compared with MHC-II molecules, MHC-I molecules bind shorter peptides between 8 and 11 amino acids.^50^ Based on artificial neural network (ANN) training methods and position-specific scoring matrix (PSSM), many peptide-MHC-I (pMHC-I) binding affinity prediction tools, such as the currently widely used tools, NetMHC and NetMHCpan, have been developed.^91–93^ Moreover, even without ANN training, the PSSM-based software called PSSMHCpan could accurately and efficiently predict the pMHC-I binding affinity. After analyzing a 10-fold cross-validation of a training database containing 87 HLA alleles and another independent dataset, Li et al. claimed that PSSMHCpan may be superior to other currently available methods; however, this finding requires verification in further research.^94^ Currently, the industry standard for predicting the pMHC-I binding affinity is NetMHCpan-4.1, though the number of candidate tumor antigens that could be identified by specific T lymphocytes remains low.^95–97^ Using mass spectrometry datasets for model selection, MHCflurry provides another choice in addition to tools based on ANNs, which have been validated to show competitive accuracy.^98^ With the development of AI, an increasing number of tools based on deep neural networks are also promising for improving the current situation, which we would discuss in the following section of the review.

The process of the formation of peptide-MHC-II (pMHC-II) is similar to that of pMHC-I, but it usually binds longer peptides up to 30 amino acids. Furthermore, the impressive diversity of the length of MHC-II-binding peptides and the “openness” of the peptide-binding groove of HLA class II, which permits the binding of a highly degenerate set of peptides, both hinder the development of competitive predictive tools.^99,100^ Therefore, the prediction of pMHC-II affinity is more challenging, and naturally, the number of available pMHC-II binding affinity prediction methods is far less than pMHC-I, such as CONSENSUS, ProPred, MixMHC2pred, MHCnuggets, NetMHCII, and NetMHCIIpan.^101–105^ As frequent updates, NetMHCIIpan may be the priority for researchers depending on its competitive performance. Nielsen et al. used the epitope dataset described by Reynisson et al.^106^ for independent validation and found that NetMHCIIpan-4.0 is much better than the other tools. However, research gaps still persist in the prediction of antigen-MHC II affinity, representing a pause in the development of the prediction of tumor antigens.

Although varying in principles, intended uses, and input and output formats, these tools are not perfect in all aspects, such as sensitivity, accuracy, and availability. Much work is needed to optimize the current tools to better predict tumor antigens to assist with follow-up vaccine design.

Considering the above information, immunogenomics technologies in the NGS era allow researchers to take full advantage of and comprehensively understand sequencing data. On the one hand, considering that conventional tools used to calculate the content of immune cells, such as flow cytometry and IHC, could only quantify a few cellular subtypes, immunogenomics technologies represented by CIBERSORT and ESTIMATE could simultaneously quantity dozens of immune cell types at a relatively lower cost with considerable convenience. On the other hand, with the development of the abovementioned sequencing technology and bioinformatic algorithms, researchers could extract maximal meaning from sequencing data to correlate genetic abnormalities with anti-tumor immunity to make predictions regarding tumor antigens, enabling the design of tumor vaccines.^107^ Thus, in the NGS era, the technological advances of immunogenomics greatly promote the development of tumor immunity research.

## Immunomics in the single-cell era

Although studies using NGS technologies to investigate tumor immunity have greatly promoted the development of oncology, the deficiencies of bulk sequencing have gradually emerged. Performed with RNA (or DNA) extracted from tissue or large cell populations, bulk sequencing may result in a dilution of the signal below the lower detection limit and average out individual cellular expression patterns, masking the reaction of a single cell.^108–111^ In addition to intra-tumoral heterogeneity (ITH) and the dramatic diversity of immune cells, numerous significant biological phenomena may be obscured by bulk sequencing in the exploration of tumor immunity.

Until recently, technological breakthroughs in single-cell-related approaches revolutionized our understanding of tumor immunity and transitioned the research level from the bulk level to the single-cell level.^112–115^ In addition to immune cells and tumor cells in the TIME, all cells in the TIME are highly heterogeneous and have unique gene expression profiles and membrane protein expression. We can utilize sequencing technologies and antigen-antibody combination reactions to reflect the features of a single cell. Here, we mainly discuss several technologies applied to the tumor immune cell repertoire and TIME spatial architecture^20,116–118^ (Table 3).

**Table 3:** Comparison of immunomics technologies at the single-cell level

Technology	Spatial	Strengths	Weaknesses
H&E	√	Simple intelligible protocol Lower cost and less time Impressive preservation of tissue morphology	Lack of specific markers Only morphological features and basophilic or eosinophilic information available
mIHC&IF	√	Highly specific marker Detailed information regarding the abundance, distribution and localization of certain substances	Spectral overlap Limited simultaneously detectable markers Time-consuming and labor intensive
Flow cytometry		Affordable and fast Machinery available in most institutes More tools available for analysis Could perform cell sorting	Spectral overlap Fluorescent spill-over Targets need to be selected carefully (biased)
CyTOF		More simultaneously detectable markers Higher accuracy without spectral overlap	Costly (both the machine and antibodies) Slower processing speed and lower sensitivity Targets need to be carefully selected (biased)
Spectral flow cytometry		Compatible with flow cytometry (both the machine and antibodies) Greatly eliminates confounding factors	Targets need to be carefully selected (biased)
Single-cell seq		Unbiased Parallel multi-omics analysis Generation of new hypotheses	Limited to nearly 10,000 cells Limited sequencing depth/coverage Costly, time-consuming and labor intensive
CODEX	√	Higher accuracy and specificity Detection of over 50 markers in a single slide	Affected by the tissue quality Accumulative structural changes Costly, time-consuming and labor intensive
IMC	√	At near-optical resolution Could be applied to biobanked tissues More simultaneously detectable markers	Lack of suitable commercial antibodies for use Comparatively lower rate of image acquisition Limited extent to which slides can be scanned Costly and only available in high-end facilities
MIBI-TOF	√	High accuracy at near-optical resolution Could be applied to biobanked tissue Indefinitely stable samples More simultaneously detectable markers	Lack of suitable commercial antibodies for use Comparatively lower rate of image acquisition Limited extent to which slides can be scanned Costly and only available in high-end facilities
Spatial transcriptomics	√	Visualization and quantitative analysis of the transcriptome with spatial resolution	Small-niche but not real single-cell sequencing Comparatively low resolution
Slide-seq	√	High spatial resolution High scalability to large tissue volumes Lower cost and better accessibility	Small-niche but not real single-cell sequencing Not suitable for analyzing multiple sections Confined to transcriptomics data
HDST	√	Higher spatial resolution than Slide-seq High scalability to large tissue volumes Lower cost and better accessibility	Small-niche but not real single-cell sequencing Not suitable for analyzing multiple sections Confined to transcriptomics data
DBiT-seq	√	Unbiased High spatial resolution multi-omics seq Compatible with different tissues High accessibility and operability	Small-niche but not real single-cell sequencing Existence of a theoretical limit of the pixel size
ZipSeq	√	Provides a complete map of live tissues May integrate with multimodal measurements	Confined to transcriptomics data Costly and only available in few facilities

*CODEX* codetection by indexing, *CyTOF* cytometry by time-of-light, *DBiT-seq* deterministic barcoding in tissue for spatial omics sequencing, *HDST* high-definition spatial transcriptome, *H&E* hematoxylin-eosin, *mIHC* multiplex immunohistochemistry, *mIF* multiplex immunofluorescence, *IMC* imaging mass cytometry, *MIBI-TOF* multiplexed ion beam imaging by time-of-flight

### Single-cell-based tumor immune cell repertoire

As a highly complex whole, the biological behaviors of tumors, including carcinogenesis, tumor progression, metastasis, recurrence, and response to therapy, all depend on the crosstalk between tumor cells and the surrounding cells in the TIME, especially the immune stromal elements.^22,119^ Therefore, characterizing the TIME and determining the cellular components could be highly beneficial for tumor immunity studies.

#### Protein-based single-cell analysis—THE KNOWN UNKNOWN

##### Polychromatic flow cytometry

Based on the physical characteristics and proteins expressed on the cell surface or within cells that are relatively unique to each cell type, flow cytometry could identify and quantify various cell types utilizing fluorescent dye-conjugated antibodies.^120^ Flow cytometry has emerged as a core tool in medical research, particularly regarding tumor immune cells.^121^ The power of multiparametric analysis to discriminate functionally and physically distinct subsets of immune cells has driven flow cytometry to the routinely used 8-parameter flow cytometer. In addition, coupled with technological advances, the design, and implementation of instruments that could measure more parameters (including fluorescent colors and physical parameters) are and could be realized, such as 30- and 50-parameter flow cytometers.^122,123^ The more parameters that can be measured by flow cytometry, the more information that can be attained from the same sample for further advanced analysis (this also enhances the difficulty of analysis and decreases the accuracy, which we would discuss below). Technological development is confined to not only improving the number of measurable parameters but also better analyzing the existing data. For example, more computational tools for preprocessing, population identification (e.g., FlowJo, FCS Express, WinMDI, and CytoPaint), clustering (e.g., DensVM, kmeans, and mclust), visualization (e.g., flowViz, ggCyto, RchyOptimyx, SPADE, Citrus, and t-stochastic neighbor embedding (t-SNE)), and sorting (e.g., fluorescence-activated cell sorting (FACS)) are available.^124–126^ However, when deciding how to optimize flow cytometry, researchers are often faced with the following dilemma: more measurable parameters with a lower accuracy or a higher accuracy with limited measurable parameters, particularly due to the overlap between the emission spectra of fluorochromes. Thus, to some extent, these disadvantages limit the application and further development of flow cytometry.

##### Cytometry by time-of-flight

Mass cytometry, which is a recent innovation in this field and is also termed cytometry by time-of-flight (CyTOF), combines flow cytometry with mass spectrometry and bridges the gap.^122,127^ Compared with traditional flow cytometry, mass cytometry labels antibodies with metal isotopes instead of fluorophores and then quantifies the signal using a time-of-flight detector, which detects at least 40 parameters and avoids the problem of spectral overlap. CyTOF has been validated as an accurate approach for performing high-dimensional analyses of tumor tissues for exploratory immune profiling and biomarker discovery.^128,129^ Chevrier et al.^130^ applied mass cytometry to successfully depict an in-depth atlas of the TIME in clear renal cell carcinoma and correlated immune compositions with clinical features, which has great clinical significance and could guide follow-up studies. Another interesting study performed by Friebel et al. creatively showed that the immune response to cancer in the brain is shaped by the cancer type. Using CyTOF, the TIME of patients with primary brain tumors and brain metastases could be mapped and differentiated according to the heterogeneous composition of tissue-resident and invading immune cells, facilitating the proper design of follow-up targeted immunotherapy strategies.^131^

Although mass cytometry theoretically allows us to detect at most 100 parameters per cell, the processing speed and throughput are limited by ion flight. After being atomized and ionized, cells are completely destroyed during preprocessing, rendering follow-up cell sorting applications infeasible.^122^ In addition, regarding measuring certain low-expressed molecular features, CyTOF may be inappropriate because of its low sensitivity.^127^

##### Spectral flow cytometry

Spectral flow cytometry is another recent technological advance that promotes the efficacy of conventional flow cytometry. Differing from mass cytometry, spectral flow cytometry still labels antibodies with fluorescent dyes but replaces classical optics and detectors with dispersive optics and novel detectors that measure the full emission spectrum.^132^ Based on the same principle, conventional flow cytometry and spectral flow cytometry maintain fairly good compatibility, particularly regarding the availability of commercial antibodies, but better eliminate confounding factors, such as spectral overlap, to improve efficiency. Along with the development of compensation technologies, spectral flow cytometry has the potential to replace polychromatic flow cytometry.^133^

Flow cytometry, mass cytometry, and spectral flow cytometry all base on binding a specific label with the corresponding cellular subgroup and identifying that label, indicating that the targets must be determined before sample acquisition. Thus, the initial targets limit the information obtained from these technologies, seriously diluting the creativeness of research findings.^127^ We believe that we can only find “THE KNOWN UNKNOWN” via these technologies. In addition, during the actual process, the expense, processing speed, and operability should all be carefully considered. For example, although mass cytometry can avoid the problem of spectral overlap, the cost of specific detectors and access to the required commercial antibodies could render the technique impractical. Finally, we believe that these three technologies are based on the expression of proteins, which may provide a relatively narrow view of the single-cell repertoire, particularly in the era of multi-omics, and urgent innovations are needed.

#### Single-cell RNA sequencing—THE UNKNOWN UNKNOWN

Fortunately, the advent of single-cell sequencing has driven the single-cell area to new heights. Based on NGS, single-cell sequencing can be divided into the following two main steps: single-cell separation and single-cell analysis.^134^ Single-cell separation, which is also called single-cell isolation, plays an indispensable role in single-cell studies, including FACS, laser microdissection, manual cell picking, random seeding/dilution, and microfluidics/lab-on-a-chip devices.^135^ Regarding single-cell analysis, genomic, transcriptomic (mainly), proteomic, and even metabolomic profiles of a single cell are unquestionable research priorities.^136–138^ No longer limited by predetermined targets as flow cytometry, an individual cell can be sequenced using the standard NGS protocol to obtain unbiased multi-omics profiling that can be used to identify “THE UNKNOWN UNKNOWN”.

Currently, the application of scRNA-seq is relatively more mature than other methods, to be our focus here. Zeisel et al. revealed cell types in the mouse cortex and hippocampus by scRNA-seq, which is a finding considered a groundbreaking discovery.^139,140^ Subsequently, research applying scRNA-seq to depict the TIME began worldwide. Tirosh et al.^141^ unraveled the ecosystem of metastatic melanoma by scRNA-seq to provide insight with implications for both targeted and immune therapies. Moreover, in human triple-negative breast cancer (TNBC), the combination of single-cell DNA and RNA sequencing also helped depict the evolutionary trajectories of chemoresistance, which provided further directions for therapies.^142^ Thus, the prospects of single-cell sequencing technologies are promising and deserve further investigation considering their scientific merit and clinical significance. Since the specific experimental protocols of single-cell sequencing have been reviewed in detail recently, we do not list them again but discuss their advantages and disadvantages in discriminating cellular components.^143–145^

Commonly, the technical noise resulting from the amplification of trace materials remains the most significant challenge. Regarding other drawbacks, considering scRNA-seq, the whole workflow contains the following five basic steps: single-cell sample preparation, whole-genome or transcriptome amplification, library preparation, sequencing, and data analysis. How to isolate a single cell and maintain its biological activity, how to address the vast technical noise introduced by amplification and improve sensitivity, how to obtain the highest amounts of measurable genes at the lowest price, and how to more efficiently analyze the data greatly raise the threshold for single-cell sequencing and limit its widespread use. Although currently, all technologies compromise coverage, sensitivity or throughput to some extent, we are still optimistic regarding the development of single-cell sequencing and expect more benchmarking studies in the future.

### Approaches used to identify the TIME spatial architecture

**Fig. 2:**
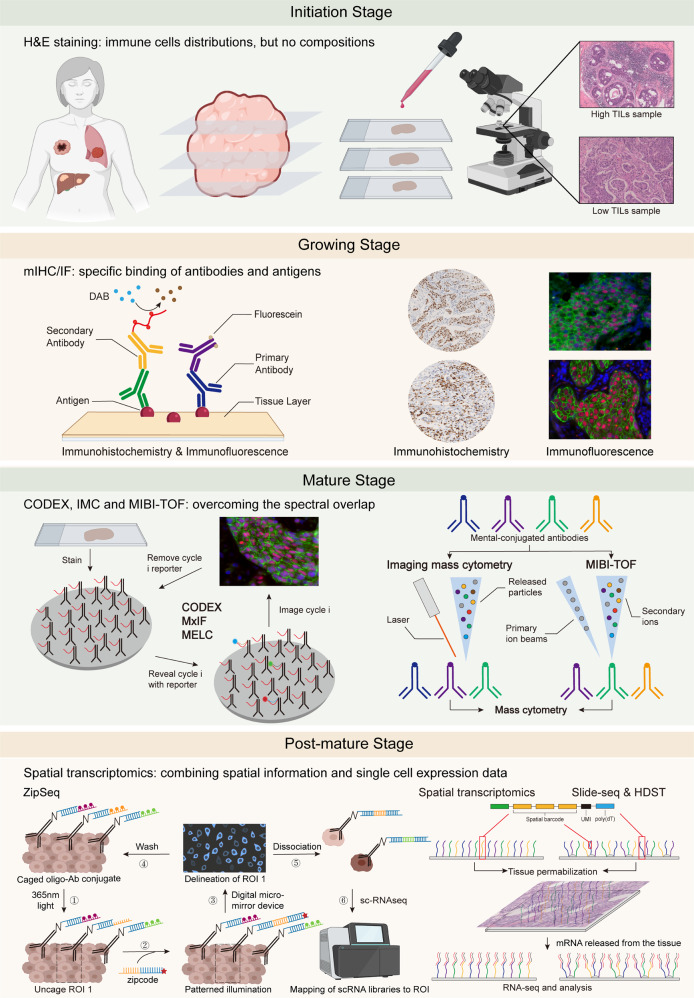
Development of single-cell spatial technologies: from germination to maturity. (1) Initiation stage: H&E staining, a conventional but significant method that clearly demonstrates the cellular and tissue structure but underperforms in the discrimination of immune cells. (2) Growing stage: The specific binding of antibodies and antigens drove the spatial technologies to a new height as represented by IHC and IF. In addition, multiplex IHC/IF technologies allow the detection of multiple markers simultaneously on a single slice, improving our understanding of the TIME spatial architecture. (3) Mature stage: Given that the spectral overlap limits the further application of mIHC/IF, utilizing dye cycling is a main optimization strategy in which only two or three antibodies are imaged by fluorescence microscopy in each cycle. Then, the fluorophores are cleaved and washed, and this cycle is repeated until all antibodies are imaged, such as CODEX, MxIF, and MELC. Also, IMC and MIBI-TOF utilize mental-conjugated antibodies to eliminate confounding factors, such as spectral overlap, and are also promising. (4) Postmature stage: Combining high-resolution spatial information with single-cell expression data, spatial transcriptomics, slide-seq, HDST, etc. explore brand-new ideas for the characterization of the spatial architecture. CODEX codetection by indexing, HDST high-definition spatial transcriptome, H&E hematoxylin-eosin, IF immunofluorescence, IHC immunohistochemistry, IMC imaging mass cytometry, MELC multiepitope ligand cartography, MIBI-TOF multiplexed ion beam imaging by time-of-flight, MxIF multiplexed fluorescence microscopy method

Studies have increasingly found that not only the components of the TIME but also the spatial architectures significantly influence anti-tumor immunity.^146^ Given that single-cell isolation is necessary for flow cytometry and single-cell sequencing, none of the single-cell technologies mentioned above can be applied to studies investigating spatial architecture. Thus, we briefly introduce several recent single-cell level spatial technologies. According to the principles, we divide the development of TIME spatial architecture approaches into the following four stages: initiation or emerging stage, growing stage, mature stage, and postmature stage (Fig. 2).

#### Initiation stage: H&E-staining

In the initiation stage, a microscopic analysis of the tissue components of an H&E-stained tumor sample slide allows pathologists to clearly differentiate the alkaliphilic nucleus and acidophilic cytoplasm of cells, providing an image of the spatial architecture.^147,148^ However, without specific markers, we can only empirically divide cells into several large subgroups, such as parenchyma cells, fibroblasts, muscle cells, and inflammatory cells, which is not suitable for characterizing the spatial architecture of the TIME.

#### Growing stage: mIHC & mIF

Then, at the growing stage, the development of immunological markers markedly improved TIME spatial architecture approaches. IHC and IF utilize fluorescent dye- or enzyme reporter-labeled antibodies targeted against certain antigens in specific cells to more precisely discriminate cell types.^149,150^ Multiplex immunohistochemistry/immunofluorescence (mIHC/IF) enables the simultaneous detection of multiple markers on a single slice, improving our understanding of the TIME spatial architecture. Unfortunately, similar to flow cytometry, mIHC/IF remains limited by spectral overlap.

#### Mature stage: CODEX, IMC, and MIBI-TOF

Entering the mature stage, codetection by indexing (CODEX), which is a multiplexed cytometric imaging approach, replaces fluorescent dyes or enzyme reporters with designed specific barcodes comprising a unique oligonucleotide sequence. The fluorescent dNTP analogs and in situ polymerization-based indexing procedure help provide an image of the slice. Interestingly, cells are stained with a mixture of all tagged antibodies simultaneously, but only two or three antibodies are imaged by fluorescence microscopy at each cycle. Then, the fluorophores are cleaved and washed, and the cycle is repeated until all antibodies are imaged. Using computational tools, all antibodies are visualized to reconstruct the multiparameter image.^151^ Hence, the accuracy of CODEX is higher than that of mIHC/IF to minimize spectral overlap. With the help of CODEX, Goltsev et al.^151^ observed many previously uncharacterized splenic cell-interaction dynamics in fresh-frozen spleen tissues from animals with systemic autoimmune disease, which is promising for enabling the systemic characterization of tissue architecture. Regarding cancer, the application of CODEX also enabled Schürch et al.^146^ to identify conserved, distinct cellular neighborhoods (CNs) and explore their correlation with clinical outcomes, re-engineering them to be compatible with formalin-fixed, paraffin-embedded (FFPE) tissue and tissue microarrays.

The multiplexed fluorescence microscopy method (MxIF) and multiepitope ligand cartography (MELC) are two other technologies that use dye cycling analogous to that in CODEX, allowing the detection of at most 100 antigens in a single sample.^152–154^ In contrast, MxIF is superior to MELC because it can provide a quantitative, single-cell, and subcellular characterization of multiple analytes in FFPE tissue and integrate histological staining with DNA fluorescence in situ hybridization (FISH) to unambiguously compare identical regions in the same sample.^154^ However, the characteristic feature of these technologies also results in a disadvantage, as follows: repeated elution and imaging could change the antigenicity of the target specimen and may cost too much time and money.

In addition, imaging mass cytometry (IMC) is another expansion of mass cytometry that is perhaps similar to the combination of IHC and mass cytometry. IMC uses laser ablation to generate particles that are carried to the mass cytometer by inserting gas and then yields a high-resolution picture of the region of interest on the slide.^155^ Notably, IMC preserves the antigen specificity and can simultaneously provide the spatially resolved analysis of 32 proteins.^156^ On this basis, Damond et al.^157^ presented a new mechanism of type I diabetes progression as follows: the loss of β cell markers and recruitment of cytotoxic T cells and T helper cells precede β cell destruction. Regarding tumors, Fisher and colleagues unraveled the spatial architecture of classic Hodgkin lymphoma to correlate LAG3 3-expressing Tr1-type Treg cells with MHC-II-negative Hodgkin lymphoma.^158^

In addition, based on analogous but more complex principles, matrix-assisted laser desorption/ionization (MALDI) mass cytometry can directly identify the distributions of proteins, lipids, metabolites, and drugs with a higher accuracy and sensitivity but lower sample requirements to identify large molecules rather than the TIME spatial architecture.^159–161^ Further application of laser ablation coupled with inductively coupled plasma mass spectrometry (LA-ICP-MS) is also limited by the laser spot size, analysis speed, and sensitivity but may be combined with IHC, which we do not discuss in detail here.^156,162^

Notably, the onset of multiplexed ion beam imaging by time-of-flight (MIBI-TOF) had an impact on this field. Compared with mIHC/IF, MIBI-TOF utilizes secondary ion mass spectrometry to image antibodies tagged with mental isotopes and can analyze at most 100 targets simultaneously with high accuracy, low spectral overlap, and no need for channel compensation.^163^ For example, a structured TIME in TNBC characterized by in situ expression of 36 proteins covering identity, function, and immune regulation at subcellular resolution in 41 TNBC patients has been revealed using MIBI-TOF.^164^ In 2019, a purpose-built mass spectrometer for MIBI analysis was also designed to further promote the application of MIBI-TOF.^165^ Combined with CyTOF, MIBI-TOF helped researchers to draw a single-cell metabolic profile of cytotoxic T cells.^166^ In addition, MIBI-TOF and IMC can both be applied to FFPE tissue sections to perform a retrospective analysis of patient cohorts whose outcome is known.

#### Post-mature stage: spatial transcriptomics

Finally, the development of spatial technologies has entered the post-mature stage. Recently, the post-genomics era started with an increasing number of sequenced model organisms and further decreases in cost. How to correlate high-resolution spatial information with single-cell expression data and whether single-cell sequencing technology can be utilized to characterize spatial architectures have remained hurdles for a long time in this era. Fortunately, spatial transcriptomics has emerged to address this issue. Making the best use of NGS, similar to spatial transcriptomics, slide-seq and high-definition spatial transcriptome (HDST) utilize a monolayer of spatially barcoded beads on a glass slide to capture mRNAs released from tissue placed on top to demonstrate spatial transcriptome mapping at the cellular level (which could be reduced to 2 µm for HDST).^166–171^ Furthermore, not confined to transcriptomics data, a recent innovation involving a microfluidic-based method, deterministic barcoding in tissue for spatial omics sequencing (DBiT-seq), enables the realization of high-spatial-resolution multi-omics sequencing in FFPE slides, revolutionizing a range of research fields.^172^ Notably, ZipSeq can label live cells in intact tissues with unique illumination and photocaged oligonucleotide “zipcodes” and then lyse tissues into individual cells for RNA-seq to broaden the scope of research.^173,174^ Using these technologies to match clustered regions with individual cells, a spatial landscape of the transcriptome can be generated. However, depending on the designed bead decoding or deterministic barcoding, the number of detected genes is limited; therefore, real spatial transcriptome sequencing has not been realized but is warranted.

Using scRNA-seq data and in situ hybridization patterns as the input, Seurat, which is a spatial map technique, uses a series of sophisticated models to infer the original spatial location of a single cell. This R package (Seurat v3) can accurately localize cellular subpopulations and has been developed into one of the standard tools that have been validated in zebrafish (*Danio rerio*).^175,176^ Andersson et al.^177^ also developed a model-based probabilistic method that performs guided deconvolution of mixed expression profiles to integrate scRNA-seq and spatial transcriptomics data and then spatially map cell types.

The field of single-cell spatial transcriptomics is greatly expanding, and the number of correlative technologies is exploding. Nonetheless, most technologies do not perform NGS or characterize the spatial architecture completely at the single-cell level but rather have tiny pixel sizes (10 µm, even 2 µm). These technologies still perform bulk analyses only for a smaller mixture of cells, possibly representing the greatest challenge faced by current single-cell spatial technologies. Thus, more single-cell technologies and related benchmarking studies are still expected for a long time.

## Immunomics and AI

With the development of computer technology, AI, i.e., an intelligence demonstrated by machines that mimic the cognitive functions performed by the human mind, such as learning and making decisions, has been applied all to various fields worldwide.^178^ In medicine, scientists and clinicians are paying extensive attention to the applications of machine learning or even more advanced deep learning in disease diagnosis, prognosis, and therapeutic response prediction.^179^ Regarding tumor immunity, AI assists clinicians in better analyzing tumor immunological features associated with the TIME and response to immunotherapy. The technological advances of AI in cancer immunity research principally involve the following aspects: (1) attenuating the workload of the manual recognition of immune infiltration on pathological slides; (2) offering an alternative technology to recognize immune cell subpopulations and spatial architectures that can be hardly distinguished by the human eye; and (3) providing a non-invasive approach to predict specific patient characteristics of the TIME and response to immunotherapy. The major theory of AI in cancer immunity research harnesses high-dimensional features or a black-box operation program to deeply excavate the characteristics of patients’ intra-tumoral immune infiltration.

### Tumor antigen prediction with deep learning methods

Concise and accurate tumor antigen prediction is necessary for the investigation and fabrication of personalized tumor vaccines. An important problem resulting in a relatively high false-positive rate is that the current tools predicting antigen presentation are mostly trained by in vitro binding affinity data, thus ignoring other factors, such as gene expression, proteasome cleavage, and transporters associated with antigen processing (TAP) transportation. Considering the aforementioned factors, a robust neoantigen prediction model that comprises reliable training data and an advanced algorithm framework is necessary.

The first step to deciphering tumor antigens is to predict abnormal peptides. In addition to the multiple developed algorithms identifying SNVs, a recently designed CN-learn tool has been designed to detect CNVs, exhibiting favorable performance.^180–182^ Regarding HLA typing, Bulik et al.^183^ generated a large integrated dataset including HLA types and HLA peptides from various types of cancer tissues and published data that could be used to train the full mass spectrometry deep learning model EDGE, which has been validated in non-small-cell lung cancer (NSCLC) patients. Two promising computational deep learning methods, MARIA and MixMHC2pred, were recently introduced, greatly increasing the MHC-II prediction accuracy. MARIA is trained using not only in vitro binding affinity data but also naturally presented MHC-II ligand detected by liquid chromatography-tandem mass cytometry (LS-MS/MS) and gene expression levels and conducts a recurrent neural network (RNN) to output a presentation score.^184^ Racle et al.^103^ developed a motif deconvolution algorithm, i.e., Modec, to train the deep learning MHC-II peptide predictor MixMHC2pred. These two deep learning methods outperformed the previously prevalent tool NetMHCIIpan, and the neoantigens predicted by both programs have been proven to stimulate responsive CD4^ +^ T cells.

### Radiomics in tumor immunity

**Fig. 3:**
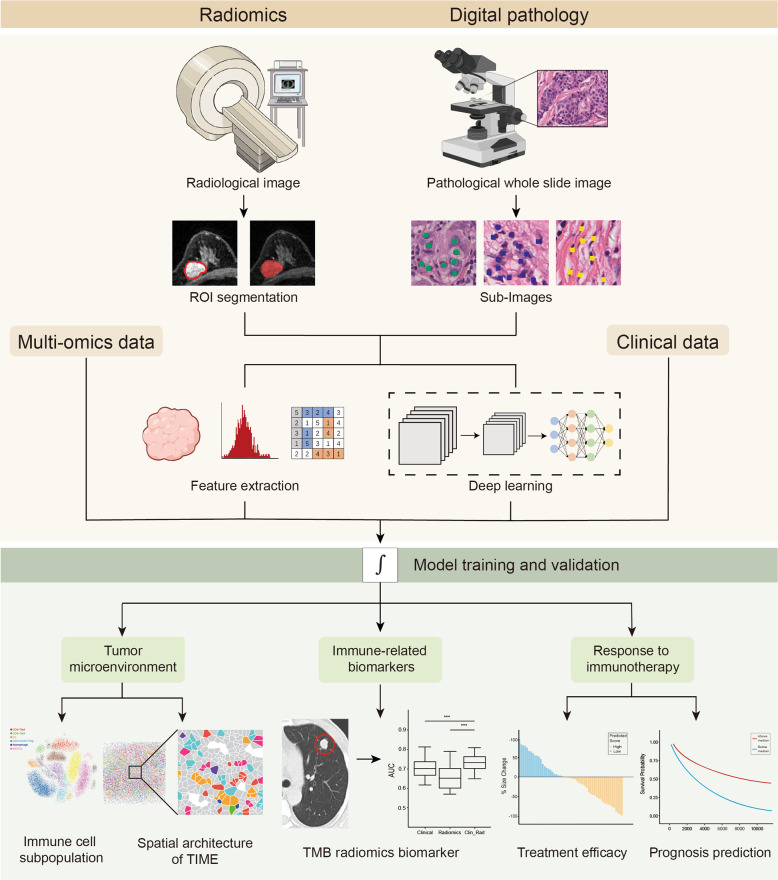
Radiomics and computational pathology in tumor immunity exploration. Radiological and pathological image-derived omics data enable the investigation of the tumor immune microenvironment (TIME) and response to immunotherapy. For a raw radiology image, regions of interest, generally representing the tumor lesion area, are segmented, while a pathological image is divided into numerous sub-images. Two methods can be applied to analyze these high-dimensional data. First, features, including but not limited to statistical features, tumor volume features, and texture features, are extracted and analyzed by professional clinicians. Alternatively, images are input into a convolutional neural network (CNN). After a complicated deep learning process, robust models are output. These radiomics or digital pathologic models could finally be established to evaluate or predict the immune index, which can be divided into three aspects. First, TIME dissection encompasses distinct immune cell subset classification and TIME spatial architecture characterization, resembling single-cell technologies and CODEX, respectively. Second, immune-related biomarkers, such as the tumor mutation burden (TMB), could be predicted. Third, response to immunotherapy and clinical outcomes could be predicted. ROI regions of interest, TIME tumor immune microenvironment, TMB tumor mutation burden

With the development of AI in medical imageology, imaging is far beyond simply a picture but a large scale of digital data. The quantitative and qualitative features extracted from regions of interest (ROIs, usually containing tumor sites) characterize tumor biological behavior and can be correlated with clinical outcomes. This process of analyzing imaging data using AI technology is radiomics.^185^ To the best of our knowledge, radiomics technology applied to tumor immunity is mainly used to identify biomarkers reflecting immune infiltration and predict the therapeutic response of ICB-treated patients (Fig. 3).

First, radiomics provides a non-invasive method to estimate immune-related biomarkers, such as the cytolytic activity score (CytAct) predicted by the deep learning of fluorodeoxyglucose positron emission tomography (FDG-PET)^186^ and the ImmunoScore of gastric cancer predicted by radiomic features.^187,188^ A tumor mutational burden radiomics biomarker (TMBRB) was also developed and outperformed the current clinical models in dividing NSCLC patients into high and low tumor mutation burden (TMB) patients who have different clinical outcomes.^189^ Interestingly, researchers have compared the alterations in the radiomic texture (DelRADx) between baseline and post-treatment CT imaging to discriminate responders from nonresponders. In particular, the relationship among DelRADx, the tumor-infiltrating lymphocytes (TILs) density, and programmed cell death ligand 1 (PD-L1) expression provides a reasonable interpretation for predicting clinical outcomes via radiomics approaches.^190^

On the other hand, radiomics applications in immunotherapy start with a radiomics signature of CD8^ +^ T cell output by a machine learning model. Imaging-related features and RNA-seq from patients in the MOSCATO clinical trial were input as the training set. This radiomics signature was confirmed to be a biomarker of the response to immunotherapy in validation cohorts. Compared with establishing connections between radiomics features and clinical responses via T cell infiltration, a radiomics biomarker was directly trained and validated using images and clinical data.^191^ This biomarker was more effective than the lesion volume in predicting the immunotherapy response and overall survival.^192^

During ICB administration, some atypical responses have been reported. One is hyperprogression (HP), which represents an unexpected accelerated progression after immunotherapy is initiated.^193,194^ Due to its poor prognosis, predictive biomarkers are desperately required. Textural characteristics and novel quantitative vessel tortuosity features were integrated to distinguish HPs from responders and nonresponders.^195^ Another response is pseudoprogression, which is defined as an increase in tumor size or newly identified lesion after treatment is initiated before a decrease in tumor size is observed. This phenomenon is due to an inflammatory pseudotumor formed by lymphocyte infiltration.^193,194^ By comparing blood, volume, and radiomics models alone or combined, a multimodality approach combining the blood biomarker LDH and radiomics features best-predicted pseudoprogression (AUC = 0.82).^196^

Altogether, radiomics technology enables the identification of changes in tumors during an early stage, stratifying patients’ sensitivity to immunotherapy and predicting their clinical outcomes via a noninvasive method. However, since most current studies are retrospective studies, these results still need to be validated in larger cohorts and prospective studies.

### Computational pathology in tumor immunity

Distinct from radiologists, pathologists are devoted to identifying histological alterations from a more microscopic perspective. H&E staining, IHC, and IF help pathologists differentiate among distinct cell populations. AI in pathology, or so-called digital pathology, provides novel insight into exploring the interaction between immune cells and tumor cells and the connection among key behaviors of cancer biology via computational analyses.

CNN-based deep learning models have been established to explore the quantification and spatial distribution of tumor infiltrating immune cells on H&E or IHC staining slides.^197–200^ In a recent study, AbdulJabbar et al. developed a deep learning framework to profile the spatial architecture of the TIME, revealing that heterogeneity in immune infiltration exists in different samples from identical patients and that prognosis depends on the number of “immune-cold” regions. In addition, evolutionary patterns, clonal neoantigens, and antigen presentation are associated with TIL distribution and the spatial complexity of the TIME.^201^ Furthermore, predictive models that incorporate the immune cell composition and spatial organization correlate with cancer prognosis, which several groups have proven in colorectal cancer, HCC, and melanoma^202–205^ (Fig. 3).

Similar to radiomics, digital pathology combined with deep learning excavates invisible information from images; however, the latter enables us to comprehend the TIME on a cellular or molecular level. Consistent with the high-dimensional imaging technology IMC and MIBI-TOF,^156,163,165^ digital pathology could be a promising approach for investigating the TIME structure and the relationship between cancer biology and therapy. More importantly, deep learning of computational pathology is a paradigm of large-scale detection, i.e., AI can analyze numerous pathological slides simultaneously. Moreover, AI maps analytical results to original slides, which provides a better visualization performance.

In this section, we focus on the AI-based excavation of medical imaging and pathological slides in the era of cancer immunity and immunotherapy. The last decade has observed great achievements in radiomics in clinical practice. In particular, radiomics exhibits potential in predicting immune infiltration and the response to immunotherapy. Although the processes of feature extraction and model training are mainly conducted manually in the current stage, we envision that in the near future, deep learning approaches in medical imaging will be highlighted in research investigating the TIME. In comparison, digital pathology primarily adopts a deep learning approach to dissect the spatial architecture. Although pathological slides involve invasive examination, they can provide more detailed immune information than imaging; thus, radiomics and digital pathology complement each other in the study of the immune microenvironment.

## Applications of immunomics in tumor immunotherapy

### Major categories of cancer immunotherapy

Cancer immunotherapy is mainly classified into the following six categories: oncolytic viruses, cytokine therapy, antibody-based therapy, ICBs, ACT, and cancer vaccines. **(1) Oncolytic viruses.** Oncolytic viruses are genetically modified viruses that enable tumor cells to attack and stimulate the immune system simultaneously. Recently, due to progress in genetic engineering, an oncolytic virus, i.e., talimogene laherparepvec (T-VEC), has been proven to benefit advanced melanoma patients and was approved by the Food and Drug Administration (FDA).^206^ **(2) Cytokine therapy.** As messengers in communication between immune cells and crucial orchestrating factors in the immune system, cytokines also have the potential to restrict tumor growth.^207^ Interleukin 2 (IL-2) was approved by the FDA for metastatic melanoma and kidney cancer as an immunotherapy regimen.^208^ Interferon (IFN) and tumor necrosis factor (TNF) are also regarded as cytokines with potential cancer therapeutic effects. **(3) Antibody-based therapy.** Monoclonal antibodies were attached to the surface marker of tumor cells and, thus, triggered an enlarged immune response or impeded signal transduction in tumor cells. At the end of the 20th century, rituximab was approved by the FDA for the treatment of non-Hodgkin’s lymphoma. Rituximab binds the CD20 molecule on immature B lymphocytes, guiding NK cells to eradicate these abnormal monoclonal tumor cells.^209^ **(4) Immune checkpoint blockades**. Immune checkpoint refers to negative costimulatory molecules expressed on immune cells and tumor cells. In the immune system, the interaction of checkpoint molecules partially offsets positive costimulatory signals to prevent the excess activation of the immune response, which is utilized by tricky tumor cells to render them capable of immune evasion.^210–212^ Consequently, blockades of such checkpoints reinforce anti-tumor immunity and yield durable therapy responses in cancer patients. **(5) ACT.** ACT involves the genetic modification of autologous lymphocytes to strengthen anti-tumor activity and reinfusion to the patient’s body.^213^ Engineering TCRs and chimeric antigen receptors (CARs) are the two types of antigen receptors designed to be expressed on T cells expanding ex vivo, redirecting T cells toward tumor cells specifically.^214^ **(6) Cancer vaccines.** Distinct from the prevention effect of conventional antimicrobial vaccines, cancer vaccines trigger the immune system to eradicate preexisting tumor cells. Effective components of cancer vaccines consist of DNAs, RNAs, proteins, and cells (e.g., tumor cells or DCs).^215^ Cell-based vaccines are classified into autologous and allogenic cell vaccines.

### Immunomics technologies: a milestone of immunotherapy

#### Principle of immunomics application in cancer immunotherapy

Immunotherapy has been one of the most important therapeutic approaches in addition to surgery, chemotherapy, and radiotherapy in multiple types of cancers. Tremendous benefits have been provided to cancer patients with this promising treatment option. Nevertheless, large numbers of patients show less response to immunotherapy. We must address two crucial missions. First, it is necessary to identify novel biomarkers to discriminate responders from non-responders to ICBs. Second, it is essential to authenticate effective targets for engineering T cells and cancer vaccines.

Immunomics technologies offer considerable insight into the microenvironment of tumors to facilitate achieving the two goals above. First, prospective biomarkers of ICBs could be identified by bioinformatics algorithms and single-cell-based technologies. With transcriptomic data, researchers enumerate the immune cell composition in the TIME and estimate the tumor purity with GSEA-based or cell deconvolution-based algorithms, such as ESTIMATE, CIBERSORT, and MCP-counter. Recent years have marked the rapid development of the identification of membrane molecules at a single-cell resolution. In addition to conventional techniques, such as IHC and flow cytometry, techniques, such as CyTOF and single-cell sequencing, permit the identification of more unraveled prognosis- or ICB efficacy-related immune cell subpopulations. Furthermore, promising techniques, such as IMC, CODEX, and MIBI-TOF, offer not only therapeutically significant cell populations but also the relative spatial distribution of distinct immune cells and tumor cells, which are potential targets or biomarkers. Radiomics technology is also able to predict the immune infiltration status in multiple cancer types and patient responses to immunotherapy.

Second, neoantigen prediction via bioinformatic algorithms and AI enables the identification of effective targets of adoptive cell therapy and cancer vaccines. Initially, HLA typing was inferred from genomics and transcriptomic data, and candidate neoantigens were predicted by mutation information and MHC-peptide binding affinity. After experimental validation (i.e., mass spectrometry, ELISpot, and MHC tetramers), selection and prioritization, ultimately determined neoantigens are utilized to generate neoantigen vaccines or neoantigen-targeted engineered T cells.^216^

#### Identifying biomarkers of ICBs for patient stratification

Although ICB is undoubtably a milestone of tumor therapy, only a proportion of patients benefit from it. Thus, therapeutic biomarkers are needed to stratify patients into sensitive and non-sensitive to ICB and guide precision medicine. These aforementioned technologies have remarkably promoted the identification of ICB-related biomarkers.

As a target of ICB, the PD-L1 expression level detected by IHC was the first discovered prediction biomarker,^217^ but several clinical trials have revealed moderate efficacy of ICB in patients with high PD-L1 expression.^218^ Other biomarkers are urgently required to fill this gap. Promising biomarkers are roughly classified into the following two categories: tumor cell-related biomarkers and immune cell-related biomarkers.

In 2014, investigators first connected TMB with the clinical survival of patients accepting CTLA-4 inhibitor therapy through WES. Subsequently, other retrospective studies also proved that high TMB correlates with a durable clinical benefit.^219–221^ Translational analyses using clinical trial cohorts of immunologically “cold” metastatic castration-resistant prostate cancer demonstrated that higher TMB is related to a better prognosis after nivolumab plus ipilimumab combination administration.^222^ Regarding the approaches used to assess TMB, due to the high cost and complicacy of WES, two surrogate NGS panels, i.e., FoundationOne CDx (F1CDx) and MSKCC Integrated Mutation Profiling of Actionable Cancer Targets (MSK-IMPACT), were approved by the FDA and validated by several prospective studies of multiple cancers.^221^

On the other hand, immune cell infiltrations, particularly TILs, play a pivotal role in the immune response. Among the determinants of the anti-tumor response of immune cells, counts, phenotypes and the spatial architecture are the three most highlighted.^22,223^ Initially, quantified by IHC or flow cytometry, the density of TILs was used to reflect the intensity of the anti-tumor response.^224,225^ Substantial studies have proven that the intensity of TILs strongly correlates with the ICB response and clinical outcomes.^224^ Furthermore, according to the number of TILs and their proximity to tumor cells, the TIME can be divided into immune-inflamed, immune-excluded, and immune-desert, which explicitly determine the response to immunotherapy and have better application.^226^

Nonetheless, a considerable proportion of TILs are only a bystander without cytolytic effects on tumor cells.^227^ To discover more ideal therapeutic and prognostic biomarkers, single-cell sequencing was used to identify more immune cell subpopulations. It has been found that TCF7^ +^ memory-like T cells improved the clinical outcomes of melanoma patients with anti-PD1 treatment, and stem-like TCF1^ +^ PD1^ +^ T cells were confirmed to be conducive to tumor control in response to ICB.^228,229^ More therapeutic and prognosis-related T cell subsets and functional status were identified.^230–233^ CyTOF was performed to compare the TIME of pre- and post-ICB-administered advanced melanoma patients. Krieg et al.^234^ identified CD14^ +^ CD16^ −^ HLA-DRhi monocytes for the prediction of the response to anti-PD-1 therapy. Furthermore, Helmink et al.^235^ leveraged CyTOF and single-cell sequencing to reveal a distinctive B cell functional status and tertiary lymphoid structure localization in a melanoma neoadjuvant ICB clinical trial cohort. In addition to adaptive immune cells, new subtypes of innate immune cells, such as macrophages, DCs, and innate lymphoid cells, were also classified by single-cell transcriptome analyses and demonstrated to influence anti-tumor immunity and prognosis^236–238^ (Table 4).

**Table 4:** Clinical significance of immunomics technologies

Category	Representative technique	Example of clinical relevance	Cancer type	Ref.
Bulk sequencing	Abnormal peptides prediction	Developing tumor neoantigen vaccines	Melanoma	^262,291^
	HLA typing		Glioblastoma	^267^
	MHC-antigen binding affinity		Melanoma, NSCLC	^292^
Conventional staining on pathological slides	Immunohistochemistry Immunofluorescence	Detecting immunotherapy biomarkers, such as PD-L1 and TILs	Multiple cancer types	^293,294^
Single-cell technologies	CyTOF	Identifying multiple prognosis-correlated T cell and macrophage phenotypes	Renal cell carcinoma	^130^
		Revealing immune cell heterogeneity between glioma and brain metastases	Brain cancer	^131^
		Discovering distinct liver TIME driving resistance to immunotherapy	Liver metastatic cancer	^295^
	MIBI-TOF	Dissecting the spatial architecture of the TIME as a promising immunotherapy biomarker	Triple-negative breast cancer	^164^
	Single-cell transcriptomics	ILCregs indicate a poor prognosis	Colorectal cancer	^238^
		TNFRSF9^ +^ Treg cells refer to a poor prognosis	Lung adenocarcinoma	^231^
		CLEC9A^ +^ DC represents a better clinical outcome	Nasopharyngeal carcinoma	^237^
		TCF1^-^PD1^ +^ T cells correlate to sensitive to immunotherapy	Melanoma	^229^
		CD11b^ +^ F4/80^+^ macrophages lead to resistance to immunotherapy	Liver metastatic cancer	^295^
		CCL22^ +^ cDC1 cells are related to sensitivity to CD40 agonist therapy	Colon cancer	^236^
		Cytotoxic CD4^ +^ T cells serve as a biomarker of responders of anti-PD-L1 treatment	Bladder cancer	^232^
Artificial intelligence	Radiomics	Radiomics signature predicts immunotherapy biomarker “CytAct”	Lung adenocarcinoma	^186^
		Radiomics signature predicts immunotherapy biomarker TMB	NSCLC	^189^
		Radiomics signature predicts chemotherapy biomarker “ImmunoScore“	gastric cancer	^187^
		Radiomics signature predicts response to immunotherapy	Multiple cancer types	^191^
	Digital pathology	Discovering the relationship between the number of immune cold regions and tumor relapse risk	Lung adenocarcinoma	^201^

*cDC* conventional dendritic cell, *CytAct* cytolytic activity score, *CyTOF* cytometry by time-of-light, *DC* dendritic cell, *HLA* human leukocyte antigen, *ILCreg* regulatory innate lymphoid cell, *MHC* major histocompatibility complex, *MIBI-TOF* multiplexed ion beam imaging by time-of-flight, *NSCLC* non-small cell lung cancer, *TMB* tumor mutation burden, Treg regulatory T cell

In addition to the components of immune cells in the TIME, the spatial organization largely influences the anti-tumor efficacy of immunotherapy. Recently, CODEX was used to image distinct cell subtypes from low-risk and high-risk colorectal cancer patients. Through a computational analysis, researchers established a CN model and then revealed different functional states in CNs and communication networks between CNs, representing the spatial heterogeneity of the TIME and correlates to clinical outcome.^146^ From another perspective, deep learning models have been used to analyze digital pathological slides to elucidate the spatial heterogeneity of tumor antigen presentation and tumor evolution.^201^ In breast cancer, researchers designed an IMC panel that enables 35 biomarkers to be labeled simultaneously, thus revealing breast cancer and connecting heterogeneity with clinical outcomes.^239^ While these emerging technologies are not mature enough, a landscape will be portrayed, and the TIME organization could be watched even from a higher dimensional perspective in the future (Table 4).

#### Predicting neoantigens for ACT therapy

ACT is an immunotherapy approach in which genetically modified or expanded autologous or allogeneic T cells are reinfused into patients to enhance anti-tumor immunity.^240^ Immunogenomics primarily functions in the identification of ideal tumor antigens in ACT therapy. Specifically, once patient NGS data are obtained, it is feasible to enter an ACT-targeted tumor antigen prediction pipeline comprising abnormal peptide prediction, HLA typing, antigen-MHC binding affinity, and neoantigen prioritization.

As the first approach of ACT, engineering TCR T cells construct tumor antigen-specific TCRs to recognize tumor-associated antigens (TAAs), such as MAGE, NY-ESO-1, or more ideal target neoantigens defined by immunogenomics data by WES and RNA-seq.^241^ chimeric antigen receptor T cells (CAR-T cells) are another approach in which T cells are armored by CAR dominantly composed of a single-chain variable fragment (scFv) from a monoclonal antibody. In contrast to TCR-engineered T cells, CAR-T cells recognize tumor antigens with an MHC-independent pattern, directly identifying and combining targeting surface molecules expressed on tumor cells.^242^ Although successful in multiple hematopoietic malignancies, the benefit of CAR-T cells in solid tumors has not been forthcoming.^243–245^

Clinical trials have been conducted to demonstrate the antitumor response of TAA-specific T cells produced by TCR engineering in synovial sarcoma, melanoma, and colorectal cancers.^246–248^ Currently, neoantigen-specific TCR-engineered T cells have not been applied clinically at bedside. However, it is gratifying that several case reports have shown the efficacy of T-cell recognition against tumor neoantigens predicted by immunogenomics in colorectal cancer, breast cancers, and cholangiocarcinoma.^249–251^ Researchers cocultured T cells with neoantigen-armored APCs and T cells to identify neoantigen-activated T cells and reinfused them back into the body. Tran et al. conducted WGS of a sample from a metastatic cholangiocarcinoma patient to identify 26 somatic mutations. Tandem minigenes composed of the mutated genes were transcribed and transfected into autologous APCs, after which the neoantigen-presenting APCs were cocultured with patient-derived TILs, eventually identifying antigen-specific CD4^ +^ Vb22^+^ T cell clones, which induced regression of epithelial cancer.^251^

Resulting from the difficulty in isolating TILs from tumor sites, peripheral blood neoantigen-recognizing T cells were isolated and proved to be identical to TILs in the immunological process.^252^ Thus, WES along with neoantigen T cell isolation has become a promising approach for promoting noninvasive cancer therapeutic strategies.

However, conventional neoantigen selection based on autologous APC and T cell coculture is limited by its low throughput, high cost, and time-consuming attributes. To eliminate these barriers, more high-throughput immunogenic neoantigen detection technologies have been developed. Li et al. established a trogocytosis-based platform in which surface marker proteins transfer from APCs to T cells when TCR and pMHC combine. Therefore, ideal neoantigens could be identified by analyzing marker protein-positive cells.^253^ Coincidently, another cell-based platform utilizing signaling and antigen-presenting bifunctional receptors was established for neoantigen identification.^254^ In these cases, cell lines expressing the predicted neoantigens and TCR replaced patient-derived APCs and lymphocytes and realized high-throughput neoantigen selection as burgeoning immunogenomics technologies.

#### Selecting neoantigens for personalized cancer vaccines

Since William Coley discovered that bacterial toxins elicited body immunity to attack tumor cells, cancer vaccines have received attention.^255,256^ Subsequently, the discovery of TAA paved the way for further investigations of tumor-specific vaccine therapeutics.^257,258^ However, targeting TAAs likely harms normal cells by autoimmunity, and anti-tumor immunity is insufficient since T cells experience negative selection in the thymus against autoantigens.^259–261^ Personalized “neoantigens” from tumor mutations are more appropriate for effective vaccine design, and personalized vaccines have achieved favorable efficacy in several clinical trials, such as GAPVAC-10 and IVAC MUTANOME.^262–269^

Immunogenomics approaches have been widely applied in vaccine development in clinical research. In general, neoantigens used to generate personalized vaccines are identified by analyzing WES and RNA-seq of tumor and normal tissues and predicting effective epitopes via algorithms, such as NetMHCpan. Through this method, for instance, four in six high-risk melanoma patients accepting vaccination were free of recurrence in 25 months, while the other two patients received ICB after recurrence and had a complete response. Furthermore, ex vivo immunological experiments indicated that polyfunctional CD4^ +^ T cells and CD8^ +^ T cells were stimulated by 60% and 16% neoantigens, respectively.^262^ Similarly, neoantigen vaccines have shown efficacy in phase Ib clinical trials of glioblastoma. Single-cell TCR analysis also suggests that antigen-specific T cells are stimulated and distributed in intracranial tumor lesions.^267^

Similar to ACT, the crucial parameter of tumor vaccine development is ideal neoantigen identification. Considerable efforts have been exerted to develop immunogenomics technology to improve the neoantigen prediction accuracy and prioritize immunogenic neoepitope selection pipelines. In a recent study, Wells et al.^270^ compiled all neoantigen prediction and selection methods and provided a brand-new candidate determination pipeline incorporating 14 immunogenic features of MHC presentation and T cell recognition. This study lays a solid foundation for promoting the efficacy of tumor vaccines and adoptive cell therapy.

## Conclusions and future directions

**Fig. 4:**
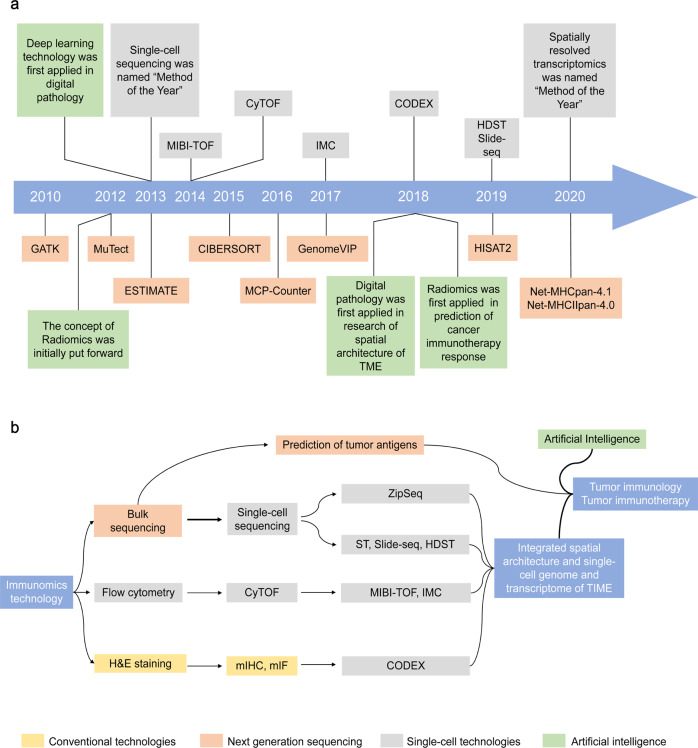
A landscape of immunomics: developmental tendency and future direction. **a** The timeline of immunomics technologies. **b** Historical development trajectory and future prospective of tumor immunomics. CODEX codetection by indexing, CyTOF cytometry by time-of-light, ESTIMATE estimation of stromal and immune cells in malignant tumors using expression data, GATK Genome Analysis Toolkit, GenomeVIP Genome Variant Investigation Platform, HDST high-definition spatial transcriptome, IMC imaging mass cytometry, MCP-counter microenvironment cell populations-counter, MIBI-TOF multiplexed ion beam imaging by time-of-flight, mIF multiplex immunofluorescence, mIHC multiplex immunohistochemistry

It is patently obvious that with the giant leap of emergent technologies in the realm of immunomics, we are now able to dissect tumor immunity at an unprecedented depth (Fig. 4). In this review, we present a picture of conventional and state-of-the-art technologies in tumor immunology along with prospects for clinical application as a reference for researchers.

In the era of bulk sequencing, methods for estimating tumor immune cells, mainly including computational algorithms, such as CIBERSORT and MCP-counter, allow us to better explore the individual infiltration pattern of tumor immune cells. Furthermore, comprising the prediction of abnormal peptides, HLA typing, and prediction of tumor antigen-MHC binding affinity, the use of immunogenomics technologies to predict tumor antigens has demonstrated credible efficacy in both preclinical and clinical studies, as represented by personalized tumor vaccines and ACT.

Moreover, it is wise to explore tumor immunity at the single-cell level considering the high diversity of immune cell subtypes and ITH. With the development of single-cell immune-related technologies, from flow cytometry and spectral flow cytometry to CyTOF, the single-cell tumor immune atlas should assist with immune cell subgroup classification to decipher components of the TIME. Regarding spatial architecture, using H&E, IHC/IF, MIBI-TOF, or spatially resolved transcriptomics, which is a crowned method of 2020, provide a high-resolution visualization of the TIME.^271^

The advent of AI also provides a new direction for the development of immunomics. Radiological and pathological image-derived omics data enable the characterization of the TIME to predict the prognosis and response to immunotherapy, indicating the potential of clinical applications with noninvasive or minimally invasive methods.

As immunomics technologies flourish, several issues should be considered for sustainable development. First, although numerous methods for quality control and improvement of the algorithm principle have been implemented, the efficacy of these technologies can be improved. In particular, regarding the prediction of tumor antigens, single-cell sequencing, and spatially resolved transcriptomics, technical noise and confounding factors hamper subsequent analyses. Second, more cost-effective, accessible, and automated technologies are expected to emerge to revolutionize the development of the discipline. Third, we also expect that researchers will fully use existing technologies to explore tumor immunity and promote clinical transformation. Utilizing advanced technologies to analyze samples from clinical trials may be a practical solution. For example, Grasso et al.^272^ showed that an increase in T cell infiltration and downstream IFN-γ signaling drive clinical responses by analyzing the CheckMate 038 study using technologies, such as NGS and immune cell quantitation, representing the regeneration of immunogenomics in the NGS era. Studies investigating tumor immunotherapy, such as tumor vaccines and ACT, should be promoted. Finally, it is necessary to develop more cancer type-specific technologies. Currently, some technologies are indeed appropriate and perform well in specific tumor types. For example, spatial single-cell technologies are suitable for solid tumors because the spatial architecture of the TIME is not involved in hematological malignancies. TCR-T cell therapy is mainly applied in melanoma, and CAR-T cell therapy performs better in hematological malignancies such as leukemia and lymphoma; neoantigen prediction technologies are suitable for these cancer types. However, as discussed above, tumor type-specific technologies are confined to hematological/solid malignancies or immune “hot” tumors in the current stage. We anticipate that further cancer type-specific technologies will emerge based on the distinctive characteristics of each cancer, greatly contributing to the development of precision oncology.

Although there is much to be accomplished, immunomics is likely to dominate the field of future tumor immunology, and its clinical value will undoubtedly dramatically promote the development of this discipline, in the field of immunogenomics, single-cell, and AI.
